# Moxibustion treatment for Parkinson’s disease: study protocol for a randomized controlled trial

**DOI:** 10.1186/s12906-023-03995-w

**Published:** 2023-06-12

**Authors:** Chunxiao Wu, Lijun Zhao, Yuelin Guo, Xiaoqian Hao, Yaohua Fan, Peipei Wu, Jiajun Han, Qinglian Li, Xiaoling Wang, Qizhang Wang, Xiaodong Luo, Meiling Zhu

**Affiliations:** 1 Shenzhen Hospital of Integrated Traditional Chinese and Western Medicine, Shenzhen, People’s Republic of China; 2 The Research Center of Basic Integrative Medicine, Guangzhou University of Chinese Medicine, Guangzhou, 510405 Guangdong Province People’s Republic of China; 3 Department of Neurology, The Second Affiliated Hospital, Guangzhou University of Chinese Medicine, Guangzhou, China

**Keywords:** Moxibustion, Parkinson’s disease, Protocol, Randomized controlled trial

## Abstract

**Background:**

Parkinson’s disease (PD) is the second most common neurodegenerative disorder and seriously affects quality of life globally. Moxibustion is widely used to treat neurodegenerative diseases in the clinic and has achieved a beneficial clinical effect. However, strict control and high-quality randomized controlled trials are still lacking. Therefore, this trial aims to evaluate the clinical efficacy and safety of moxibustion in patients with PD and preliminarily explore the underlying mechanism.

**Methods:**

This is a randomized, single-blind and placebo-controlled trial design in which 70 eligible participants will be randomly divided into a moxibustion group and a sham moxibustion group. Baihui (DU20) and Sishenchong (EX-HN1) are selected for both groups. The treatment will be performed for 30 min per session, two sessions a week for 8 weeks. The mean change in MDS-UPDRS scores (including MDS-UPDRS II, III subscale scores and total scores) from baseline to the observation points will be the primary outcome. The secondary outcomes will include scores on the Parkinson’s Disease Questionnaire-39 (PDQ-39), Fatigue Severity Scale (FSS), Parkinson Disease Sleep Scale (PDSS), Montreal Cognitive Assessment (MoCA), and Self-Rating Depression Scale (SDS) as well as the Wexner constipation score. All the above outcomes will be assessed at 4 and 8 weeks. Laboratory blood biochemical analysis and functional magnetic resonance imaging (fMRI) will be conducted at baseline and at the end of treatment to explore the potential mechanisms of moxibustion in regulating PD.

**Discussion:**

In conclusion, the results of this trial will reveal whether moxibustion is effective for treating motor and nonmotor symptoms in PD. This trial will also preliminarily explore the underlying mechanism of the regulatory effect of moxibustion in PD, which will contribute to providing a theoretical basis for the treatment of PD.

**Trial registration:**

ClinicalTrials.gov ChiCTR2000029745. Registered on 9 August 2021.

## Background

Parkinson’s disease (PD) is characterized by movement slowness, tremor, rigidity, and even worse walking disorder [1]. According to the latest Global Burden of Parkinson's Disease Study, approximately 6.1 million people suffered from PD globally in 2016, and PD became the second most common neurodegenerative disorder and the fastest-growing disease that seriously affected the quality of life, resulting in 211296 deaths globally in 2016 [2]. The guidelines of PD indicate that the current first-line treatments for controlling PD symptoms mainly include medicine (levodopa, dopamine agonists, MAO-B inhibitors and COMT inhibitors) and surgery (deep brain stimulation (DBS)) [3, 4]. These treatments can effectively relieve motor symptoms for most PD patients. However, these treatments have a shortened honeymoon period and easily induce motor complications (dyskinesia, wearing-off, on and off, etc.) and other side effects that impact the use of these treatments and lead to a poor quality of life for PD patients, especially those taking high-dose and long-term antiparkinsonian drugs [5, 6]. Therefore, many researchers and patients seek complementary treatments to solve these problems. Among complementary therapies, moxibustion is a treatment that ameliorates PD symptoms and could reduce the side effects associated with the clinical use of antiparkinsonian medicine [7].

Moxibustion, a therapy that combines heat and moxa aroma radiation, focuses on acupuncture points to cure the disease [8]. Our previous studies indicated that moxibustion could reduce the motor symptoms of PD model behavior and exert a protective effect on dopamine neurons. The potential mechanism by which moxibustion regulates PD might involve enhancing the antiferroptotic defense effect of dopamine neurons [9, 10]. However, present studies mostly rely on preclinical research, and clinical trials concerning the use of moxibustion to treat PD are still lacking. From the clinical observation, moxibustion is widely used in neurodegenerative diseases such as Alzheimer's disease and PD in the clinic and has also achieved a beneficial clinical effect. However, strict control and high-quality randomized controlled trials with a low risk of bias are still needed to provide more robust evidence.

Therefore, we designed a randomized, single-blind and placebo-controlled study to evaluate the clinical efficacy and safety of moxibustion in patients with PD and to explore the underlying mechanism of the effect of moxibustion on PD by conducting blood biochemical analyses and functional magnetic resonance imaging (fMRI). The results of this study will provide more basic evidence for the clinical use of moxibustion for treating PD.

## Methods/design

### Study design

**Fig. 1:**
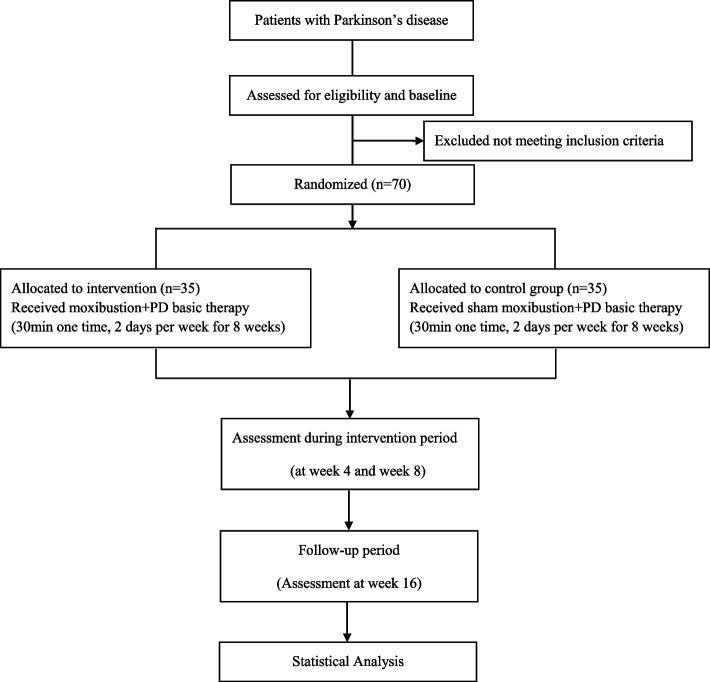
Flow chart of study design

**Fig. 2:**
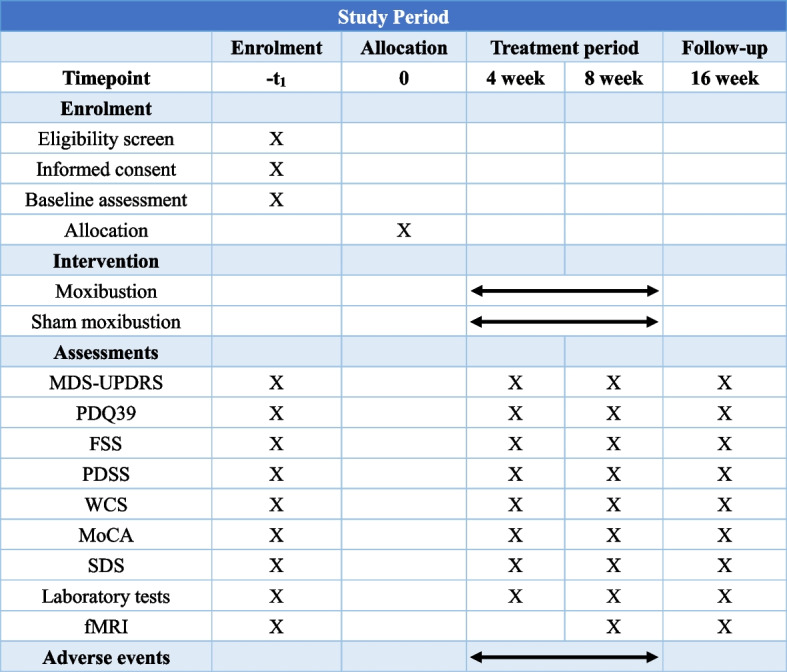
Study schedule of enrollment, intervention and assessment time points. MDS-UPDRS, MDS-Unified Parkinson’s Disease Rating Scale; PDQ39, Parkinson’s Disease Questionnaire-39; FSS, Fatigue Severity Scale; PDSS, Parkinson Disease Sleep Scale; WCS, Wexner constipation score; MoCA, Montreal Cognitive Assessment; SDS, Self-Rating Depression Scale

This is a randomized, single-blind and placebo-controlled trial to evaluate the efficacy and safety of moxibustion in patients with PD. Seventy patients who have been diagnoses with Parkinson’s disease will be randomly assigned to the moxibustion group or a sham moxibustion group at a 1:1 ratio. This protocol has been registered at the China Clinical Trial Registry (No. ChiCTR2000029745). The flowchart of this study design is shown in Fig. 1, and the specific schedule of screening, intervention allocation and assessments are summarized in Fig. 2.

### Recruitment

Participants with a PD diagnosis will be recruited by doctors from the Parkinson disease clinic at the Shenzhen Hospital of Integrated Traditional Chinese and Western Medicine. Recruitment advertisements will be posted on the hospital, public places, network, and WeChat platforms to ensure that a sufficient sample size is obtained.

### Eligibility criteria

#### *Inclusion criteria*:

1. Participants are aged between 30 and 80 years old;
2. Fulfilment of the diagnostic criteria for Parkinson's disease in China (2016 version) and Hoehn-Yahr (H-Y) stage < 3 [11]
3. Participants volunteered to participate in the trial and sign the informed consent

#### *Exclusion criteria*:

1. Other secondary Parkinson's syndrome and Parkinson's superimposed syndrome
2. Patients with dementia and mental disorders who are unable to communicate and cooperate;
3. Participants who have a history of severe cerebrovascular or brain trauma or brain surgery;
4. Complications with severe heart, lung, liver, kidney and other systemic organic diseases, endocrine diseases and metabolic disorders;
5. Participants who allergies to moxibustion treatment;
6. Participants who have undergone DBS surgery;
7. Patients who are participating in other clinical trials;
8. Other reasons that make a patient not suitable for the clinical trial as judged by the clinicians.

### Randomization and allocation concealment

In this trial, random numbers will be generated using SPSS by a researcher coordinator who is not in contact with the participants. Then, the random numbers will be sealed in sequentially numbered, opaque envelopes and delivered to clinicians. Another clinician in the clinic center will assign the patients who meet the inclusion criteria and sign the informed consent to the moxibustion or sham moxibustion groups based on the contents of the envelopes.

### Blinding

Practitioners could not be blinded because of the unique characteristics of moxibustion intervention. However, the participants will be blinded to the moxibustion and sham moxibustion group allocation. The participants will be allocated to the individual isolated treatment rooms, avoiding communication with each other. To ensure the reliability of the results, outcome assessors, personnel responsible for data collection and data statistics will be blinded to the group information and specific allocation in the entire trial.

### Intervention

The basic medicine treatment of PD in this trial will follow the principles of clinical guidelines and will not be strictly standardized because of the complexity and individualized clinical manifestations of PD. However, the dosage of the medicine taken will be recorded and cannot be changed casually.

#### Treatment group

Participants in each group will receive moxibustion and sham moxibustion intervention on Baihui (DU20) and Sishenchong (EX-HN1). In true moxibustion therapy, a moxa stick is ignited at the tip and positioned with a moxibustion supporting apparatus, and the burning tip of the moxa stick is positioned 3 cm above the corresponding acupoints. The intolerable and comfortable temperature without any burning pain above the acupoints will be controlled at 40-43 °C by a temperature thermometer. The treatment will last for 30 min per session, two sessions a week for 8 weeks (16 times). Follow-up will be conducted at week 16.

#### Control group

In the control group, participants will receive sham moxibustion treatment at the same acupoints as the true moxibustion treatment used. The nonmoxa floss stick will be ignited and positioned 8-10 cm above the acupoints. However, the temperature above the acupoints will be maintained at 37 °C, and patients will not receive any warm therapy at the acupoints. The treatment procedure and treatment duration of the sham moxibustion group will follow those of the treatment group.

### Outcome measures

The mean change in MDS-UPDRS scores (including MDS-UPDRS II, part III subscale, total score) from baseline to the observation points will be the primary outcomes. Scores on the Parkinson’s Disease Questionnaire-39 (PDQ-39), Fatigue Severity Scale (FSS), Parkinson Disease Sleep Scale (PDSS), Montreal Cognitive Assessment (MoCA), and Self-Rating Depression Scale (SDS) as well as the Wexner constipation score will be regarded as secondary outcomes and will be assessed at 4 and 8 weeks. All initial assessments will be followed up at week 16.

### Laboratory tests

Laboratory tests of serum iron, transferrin, ferritin, unsaturated iron binding capacity, malondialdehyde (MDA), and glutathione (GSH) will be collected from the venous blood at the Shenzhen Hospital of Integrated Traditional Chinese and Western Medicine. The tests will be conducted at baseline, 4 weeks, and 8 weeks and during the follow-up period.

### Functional magnetic resonance imaging (fMRI)

Functional brain imaging measuring and mapping brain activity by detecting blood oxygen level-dependent (BOLD) signal changes in different brain regions will be carried out with magnetic resonance imaging (MRI) at the Department of Radiology of Shenzhen Hospital of Integrated Traditional Chinese and Western Medicine. This test will be performed at baseline, immediately after treatment, and at the endpoint of the treatment and follow-up periods.

### Adverse events

Any possible adverse events, including skin burns, blisters, itching and respiratory symptoms that may be relevant to moxibustion treatment, will be recorded in the case report form. Other unrelated adverse events of moxibustion interventions will also be recorded and resolved by the researchers. Adverse events of both groups will be recorded throughout the trial duration, including the time, symptoms, degree, treatment group and the measures taken.

### Data monitoring and quality control

Data will be collected using paper case report forms (CRFs) and stored in a securely locked computer according to the preapproved trial protocol, and the data will be managed and monitored by the independent third party, the ethics committee of the Shenzhen Hospital of Integrated Traditional Chinese and Western Medicine. The committee will examine the original CRF, monitor the quality of the data, and verify that the study follows the principles of the trial protocol.

To ensure the quality of the trial, all researchers will be required to complete a training course of every step of this trial, including trial design, patient inclusion and exclusion criteria, intervention methods, outcome measures and records of the paper CRF. All practitioners have at least 3 years of clinical experience with moxibustion therapy and are qualified as doctors of Traditional Chinese Medicine. To improve the compliance of the participants, our researchers will contact the subjects to remind them to receive treatment or assessment in a timely manner up to 3 times via telephone. For the subjects who fail to continue participating in the clinical trial or follow-up, we will fully record the reasons and outcomes.

### Sample size calculation

A previous study explored relevant moxibustion plus basic medicine treatment versus placebo plus basic medicine treatment for PD. We extracted effective rates of 93.3% in the moxibustion group (p1) and 63.3% in the placebo group (p2) [12]. Based on these values, we calculated the sample size using the following formula: *n* = 2(u_a_ + u_b_)^2^_*_p(1-p)/(p_1_-p_2_)^2^, *p* = (p_1_*n_1_ + p_2_*n_2_)/(n_1_ + n_2_). The significance level is 0.05, and the probability values of the first type of error (u_a_) and second type of error (u_b_) are 1.96 and 0.842, respectively. The dropout rate is 15%. The estimation indicated that 35 patients were required for each group, and the total sample size was 70 patients.

### Statistical analysis

Statistical analysis of the data will be conducted by researchers blinded to group assignments using SPSS (version 25). Intention-to-treat analysis will be performed for all assigned participants. The missing data will be imputed by the last observation carried forward (LOCF) method. All included data will be first tested to determine whether they meet the normality test. If data conformed to a normal distribution, the mean ± standard deviation will be used. Otherwise, data will be expressed as M (P25-P75). An unpaired t test will be used when the data meet a normal distribution; otherwise, a nonparametric test (Mann–Whitney U) will be used to analyze comparisons between the moxibustion group and the control group. A P value less than 0.05 will be considered statistically significant.

## Discussion

The treatment of Parkinson’s disease has attracted increased attention from doctors and researchers in recent years. Although dopaminergic drug therapy is effective for controlling motor symptoms, side effects and motor complications are often induced by taking these medicines [13, 14]. Therefore, combining alternative therapies may be helpful to solve these problems. Moxibustion, as a traditional Chinese medical therapy, is beneficial for ameliorating motor and nonmotor symptoms to some extent based on clinical observations. However, high-quality trials and evidence concerning the efficacy of moxibustion for treating PD are still lacking. The objective of this trial is to confirm the effectiveness and safety of moxibustion in patients with PD and provide evidence for physicians to better improve clinical effectiveness in treating PD.

In this study, we selected the early stage of PD as our research objective because our previous preclinical studies indicated that moxibustion was effective for reducing the death of dopaminergic neurons and improving motor behavior in the early stage of PD [9, 10]. From the point of view of the clinic, the earlier the moxibustion intervention is implemented, the better the effect will be. The later stage of PD is always accompanied by severe damage and loss of dopamine neurons, and any intervention in this stage will be hard to remedy and attain satisfactory symptomatic control [15]. Therefore, to obtain a better clinical effect and observe significant group differences, we only included patients with early-stage PD.

The acupoints we will choose in these trials are DU20 and EX-HN1. Stimulating DU20 is vital to connect different parts of the brain and regulate brain function [16]. Previous reviews concerning acupoint selection for treating Parkinson's disease also demonstrated that DU 20 has been frequently applied in PD and is vital for alleviating PD symptoms [17, 18]. Our previous animal studies suggested that moxibustion at DU20 suppresses 6-OHDA-induced nigrostriatal dopaminergic cell death in a rat PD model [9, 10]. Other studies also indicated that stimulating DU20 protected dopaminergic neuronal cells from further injury and could modulate the cerebello-thalamo-cortical circuit to control PD tremor symptoms [19, 20]. EX-HN1 is located around DU20, which has a similar effect as DU20. In addition, combining EX-HN1 and DU20 will strengthen the synergistic effect for treating PD based on the theory of traditional Chinese medicine. Previous studies also suggested that moxibustion at EX-HN1 and DU 20 could protect dopamine neurons and reduce oxidative stress injury of the nigrostriatal system in rats with Parkinson's disease [21]. Therefore, we selected DU20 and EX-HN1 as our moxibustion acupoints.

Because moxibustion is a nonmedical therapy, it is difficult to apply the blinding method. The therapeutic function of moxibustion mainly contributes to the moxa material and heat stimulation. Previous methods commonly control one factor (used a base of the sham pillar or kept a far distance from the acupoints to isolate the heat stimulation) as sham moxibustion intervention [22, 23]. Another study only used nonmoxa material for sham moxification [24]. Most of these previous methods of sham moxibustion could only control one moxibustion effect factor. In this trial, we will try to control both heat and material factors that play a role in moxibustion. We set the distance of placebo moxibustion to 8-10 cm and kept a constant temperature at 37 °C, which will minimize heat stimulation and make participants feel warm, thereby creating a blinding effect [25, 26]. We also used Artemisia argyi as the sham material moxa, which might not have any treatment effect based on previous studies [24]. The sham moxibustion will maintain the same appearance as the moxa stick, and this method will make it difficult for the participants to distinguish the sham control or true moxibustion that will help to blind the participants. However, the blinding of the practitioners will be difficult to achieve because the smell, smoke and distance of the sham stick are different from those of the true moxibustion group. A third independent assessment of the treatment effect will help to overcome this limitation.

In this trial, we will estimate the overall effect of moxibustion on motor function and nonmotor function in PD to better understand and confirm the treatment effect of moxibustion in PD. In addition, we also explored the potential mechanism by which moxibustion regulates PD, which mainly involves the iron metabolism indicators that we verified in preclinical experiments [9, 10]. Previous studies also indicated that the pathogenetic mechanism of PD might be related to the ferroptosis of dopaminergic neurons [27, 28]. Therefore, we explored the potential mechanism of moxibustion in PD based on the ferroptosis mechanism in this trial. In addition, we will compare the BOLD signal changes in different brain areas using fMRI between the moxibustion and sham moxibustion groups to locate and quantify specific brain functional area changes. We attempted to directly observe the regulatory effect of moxibustion on the brain function of Parkinson's disease patients.

However, there are still limitations to this trial. First, although we tried to improve the blinding method in this protocol, due to the nature of moxibustion, genuine blinding of practitioners is difficult to achieve. This may have introduced performance bias and detection bias to an extent. Second, our study is only a single-center trial in one hospital in China, and whether these results may be able to generalize significance and applicable for other patients in other areas is still unclear.

In conclusion, the results of this trial will answer whether moxibustion is effective for treating PD in motor and nonmotor symptoms and provide a reliable and evidence-based protocol as an alternative selection for physicians to treat PD. This trial will also preliminarily explore the potential mechanism of the regulatory effect of moxibustion in PD, which will contribute to providing a theoretical basis for the treatment of PD.

## Trial status

This trial is currently in the recruitment phase. Patient recruitment started in October 2021 and is expected to finish at the end of 2023.

## Data Availability

The original contributions presented in the study are included in the article/Supplementary Material, further inquiries can be directed to the corresponding author/s.

## Abbreviations

PD: Parkinson’s disease
DU20: Baihui
EX-HN1: Sishenchong
PDQ-39: Parkinson’s disease questionnaire-39
FSS: Fatigue severity scale
PDSS: Parkinson disease sleep scale
MoCA: Montreal cognitive assessment
SDS: Self-rating depression scale
fMRI: Functional magnetic resonance imaging
MDA: Malondialdehyde
GSH: Glutathione
BOLD: Blood oxygen level-dependent
MRI: Magnetic resonance imaging
CRFs: Paper case report forms
